# Willingness to take fish-oil supplementation among hemodialysis patients: a multicenter survey following the PISCES trial

**DOI:** 10.1093/ckj/sfag269

**Published:** 2026-08-12

**Authors:** Janosch Niknam-Saeidi, Sebastian Mussnig, Anna Zabel, Luis Naar, Dilara Gülmez, Frederik Haupenthal, Haris Omic, Matthias Lorenz, Roland Edlinger, Markus Pirklbauer, Ingmar Waller, Alexander H Kirsch, Emanuel Zitt, Oliver Helk, Farsad Eskandary, Joachim Beige, Yuri Battaglia, Margriet I Hazenberg, Nienke A Manson, Casper F M Franssen, Sabine Horn, Daniel Cejka, Manfred Hecking

**Affiliations:** Division of Nephrology and Dialysis, Department of Medicine III, Medical University of Vienna, Vienna, Austria; Division of Nephrology and Dialysis, Department of Medicine III, Medical University of Vienna, Vienna, Austria; Klinik Landstraße, Vienna Health Association (Wiener Gesundheitsverbund), Vienna, Austria; Division of Nephrology and Dialysis, Department of Medicine III, Medical University of Vienna, Vienna, Austria; Department of Epidemiology, Center for Public Health, Medical University of Vienna, Vienna, Austria; Division of Nephrology and Dialysis, Department of Medicine III, Medical University of Vienna, Vienna, Austria; Division of Nephrology and Dialysis, Department of Medicine III, Medical University of Vienna, Vienna, Austria; Dialysezentrum Wien-Donaustadt, Wiener Dialysezentrum GmbH, Vienna, Austria; 3rd Department of Medicine with Metabolic Diseases and Nephrology, Klinik Hietzing, Vienna Health Association (Wiener Gesundheitsverbund), Vienna, Austria; Department of Internal Medicine IV – Nephrology and Hypertension, Medical University of Innsbruck, Innsbruck, Austria; Dr Waller Dialysis Institute (Dialyseinstitut Feldbach), Feldbach, Austria; Clinical Division of Nephrology, Department of Internal Medicine, Medical University of Graz, Graz, Austria; Division of Nephrology, Dialysis and Hypertension, Department of Internal Medicine III, Landeskrankenhaus Feldkirch, Feldkirch, Austria; Vorarlberg Institute for Vascular Investigation and Treatment (VIVIT), Feldkirch, Austria; Division of Nephrology and Dialysis, Department of Medicine III, Medical University of Vienna, Vienna, Austria; Division of Nephrology and Dialysis, Department of Medicine III, Medical University of Vienna, Vienna, Austria; Kuratorium für Dialyse und Nierentransplantation (KfH), Neu-Isenburg, Germany; Department of Medicine, University of Verona, Verona, Italy; Department of Nephrology, University Medical Center Groningen, Groningen, the Netherlands; Department of Nephrology, University Medical Center Groningen, Groningen, the Netherlands; Department of Nephrology, University Medical Center Groningen, Groningen, the Netherlands; Department of Internal Medicine, Landeskrankenhaus Villach, Villach, Austria; Department of Internal Medicine III – Nephrology and Hypertension, Transplantation Medicine and Rheumatology, Ordensklinikum Linz Elisabethinen, Linz, Austria; Institute for Physiology and Pathophysiology, Johannes Kepler University Linz, Linz, Austria; Division of Nephrology and Dialysis, Department of Medicine III, Medical University of Vienna, Vienna, Austria

To the Editor,

The PISCES trial recently reported that, among patients receiving maintenance hemodialysis, daily supplementation with 4 g fish oil (2.4 g n-3 polyunsaturated fatty acids) reduced the rate of serious cardiovascular events [0.31 vs 0.61 events per 1000 patient-days; hazard ratio 0.57, 95% confidence interval (CI) 0.47–0.70] [1]—a notable positive signal in a population where established cardioprotective strategies have largely failed [2]. Yet any benefit depends on patients’ willingness to take fish oil, and the over-the-counter supplements corresponding to the PISCES intervention are generally not reimbursed. We therefore surveyed willingness to adopt PISCES-like fish-oil supplementation among Austrian hemodialysis patients.

Between January and February 2026, members from the Hemodialysis Working Group of the Austrian Society of Nephrology distributed paper questionnaires at nine dialysis centers spanning university, regional, and peripheral units; the instrument was informed by Sekhon’s framework of intervention acceptability [3]. Willingness to take 4 g fish oil daily if recommended by the treating nephrologist was assessed under three cost scenarios—full reimbursement, partial self-payment (€20/month), and full self-payment (€40/month)—on a four-point forced-choice scale, dichotomized as willing versus unwilling. Attitudinal factors were rated on five-point Likert scales and examined for their association with willingness using univariable logistic regression. A detailed description of the methods and the full questionnaire is provided in the Supplementary material (Item S1 and Appendices S1 and S2).

Analyses included 525 questionnaires. Patient characteristics are provided in Table S1. Under full reimbursement, 79.4% (95% CI, 75.8–82.7) were willing to take fish oil if recommended by their nephrologist. Willingness decreased to 56.3% (95% CI, 52.0–60.5) under partial self-payment and to 38.1% (95% CI, 34.1–42.4) under full self-payment (Fig. 1 and Table S2). In mixed-effects logistic regression models accounting for repeated responses, partial and full self-payment were associated with lower odds of willingness than full reimbursement [odds ratio (OR) 0.03, 95% CI 0.01–0.07; and OR 0.003, 0.001–0.010; both *P *< .001], with consistent results in an ordinal sensitivity analysis (Table S3).

**Figure 1:**
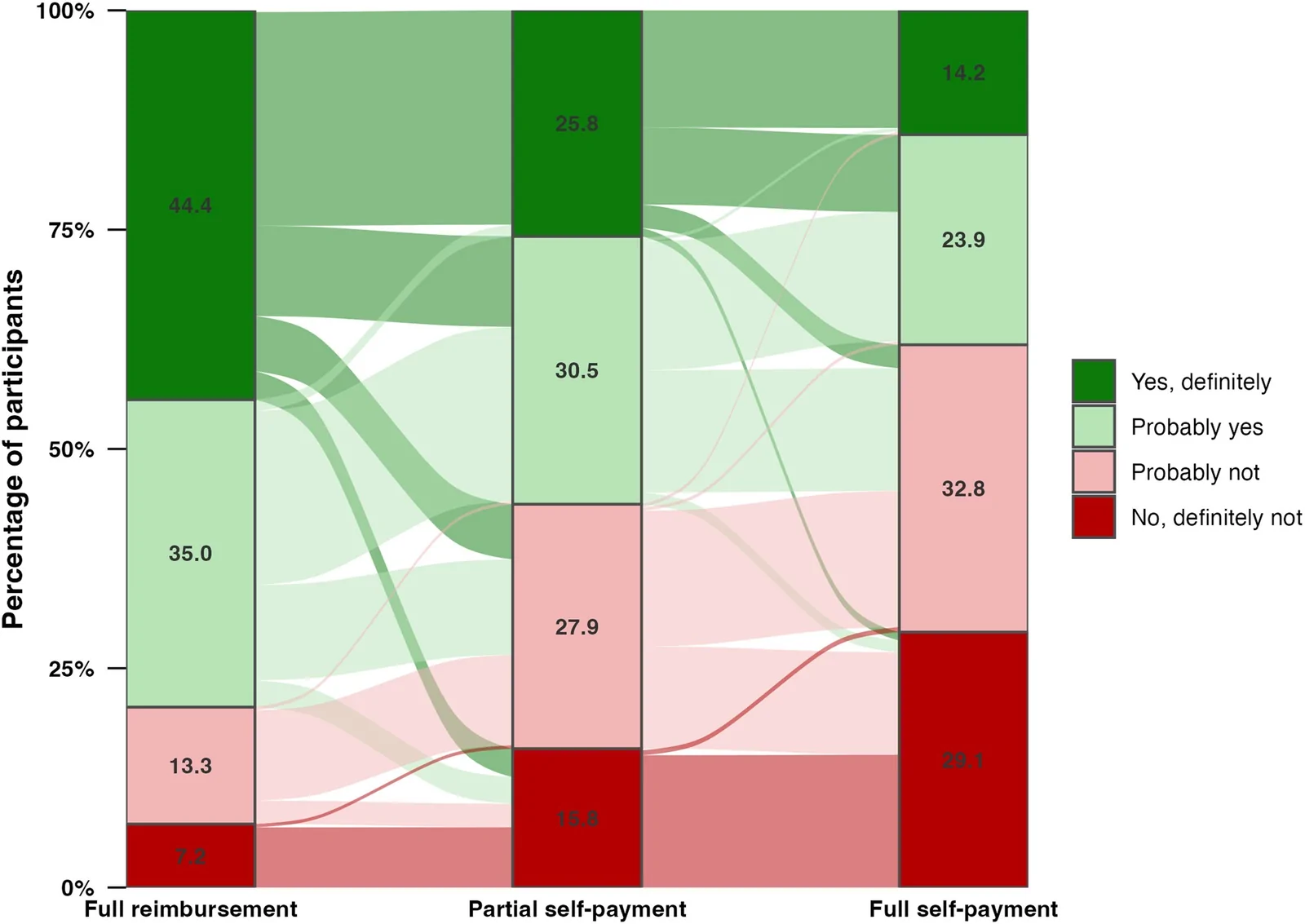
Distribution and individual shifts in willingness to take fish-oil supplementation across different cost scenarios. Legend to Fig. 1: Alluvial plot illustrating changes in individual responses regarding willingness to take fish-oil supplementation (4 g daily) under three cost scenarios: full reimbursement, partial self-payment, and full self-payment. Each vertical bar represents the distribution of response categories within a given scenario (“Yes, definitely,” “Probably yes,” “Probably not,” and “No, definitely not”). Numbers displayed within the bars indicate the percentage of participants in each response category within the respective cost scenario. Flows connecting categories across scenarios represent within-participant transitions. The width of each flow corresponds to the percentage of participants exhibiting the respective response shift.

Willingness was also shaped by attitudinal factors. Higher expected effectiveness of fish oil (OR 2.97 per point, 95% CI 2.35–3.74), greater trust in the treating nephrologist (OR 2.11, 1.78–2.51), and higher perceived cardiovascular risk (OR 1.56, 1.31–1.86) were associated with greater willingness, whereas perceived pill burden (OR 0.72, 0.62–0.84) and concerns about side effects (OR 0.68, 0.58–0.80) were associated with lower willingness (all *P *< .001; Tables S4–S6 and Figs S1, S2, and S4). Demographic and clinical characteristics showed no meaningful associations (Fig. S3).

These findings carry two messages for translating the results of the PISCES trial into practice. First, adoption will depend on financing: even modest out-of-pocket costs—plausibly a real burden for many dialysis patients—roughly halved willingness, consistent with evidence that cost drives non-adherence in chronic kidney disease [4]. Second, willingness was associated with modifiable attitudinal factors that can be addressed. Trust in the nephrologist and perceived effectiveness were the strongest positive levers, while pill burden, which is already high in this population [5], and concerns about side-effects weighed against willingness. These issues, however, could be targeted by patient-centered communication.

Our study is limited by its reliance on stated rather than actual behavior, discrete cost scenarios instead of formal preference elicitation, an Austrian-only sample without individual-level socioeconomic data, and variable participation (median 58.8%, range 20.8%–92.5%). Nonetheless, to our knowledge this is the first assessment of patient willingness to adopt fish-oil supplementation in dialysis following the PISCES trial. We conclude that whether the promising fish-oil intervention actually reaches patients will depend not only on its efficacy, but on reimbursement decisions and on how successfully nephrologists will communicate its value to patients and payers.

## Supplementary Material

sfag269_Supplemental_Files

## Data Availability

The data underlying this article are available in the article and in its online Supplementary material.
