# Supplementary material for: Willingness to take fish-oil supplementation among hemodialysis patients: a multicenter survey following the PISCES trial
Source: Clin Kidney J. 2026 Aug 12;19(9):sfag269. doi: 10.1093/ckj/sfag269 (PMC13542717; doi:10.1093/ckj/sfag269)
Supplement: Supplementary file 1 [file sfag269_supplemental_files.zip › 2026-08-04_Fish-Oil-Survey_Supplements.docx]

**Supplementary material**

**Item S1: Supplementary Methods**

Study design, study population and ethics approval

This study is a retrospective analysis of anonymized survey data collected by the Hemodialysis Working Group of the Austrian Society of Nephrology (ÖGN) between January and February 2026. The survey was conducted among adult patients receiving maintenance HD at participating centers across several Austrian federal states. Approval for this retrospective study was obtained from the Ethics Committee of the Medical University of Vienna (ethics committee number 1074/2026).

Questionnaire Development and Structure

The questionnaire was developed by the Hemodialysis Working Group of the ÖGN to assess patient perspectives on daily supplementation of fish oil in the dialysis setting. Its conceptual structure was informed by the theoretical framework of acceptability of healthcare interventions described by Sekhon et al. [18], which emphasizes cognitive and emotional responses to an intervention, including perceived effectiveness, perceived burden, and opportunity costs. The questionnaire consisted of five thematic sections, including (i) information and consent, (ii) brief information on fish oil capsules, (iii) willingness to take fish oil capsules under different cost scenarios, (iv) attitudinal factors related to perceptions and beliefs and (v) sociodemographic and clinical characteristics.

Willingness to take fish oil capsules was assessed under three hypothetical cost scenarios: full reimbursement (no out-of-pocket cost), partial self-payment (€20/month), and full self-payment (€40/month). The €40 level was anchored to the current monthly out-of-pocket price of over-the-counter n-3 PUFA supplements available in Austria that deliver a daily n-3 PUFA dose comparable to that used in PISCES (2.4 g), and €20 was chosen as an intermediate, partially subsidized level. As the PISCES intervention corresponds to such an over-the-counter supplement rather than a licensed medicinal product, self-payment reflects the default real-world condition in this setting.

Responses were recorded using a four-point forced-choice format (“Yes, definitely”, “Probably yes”, “Probably not”, “No, definitely not”). A neutral midpoint was deliberately omitted to reduce central-tendency bias and satisficing behavior and to encourage respondents to make a clear decision regarding willingness [19].

Attitudinal factors relevant to willingness - perceived CV risk, expected effectiveness of fish oil, trust in the treating physician, perceived pill burden and concerns about potential side effects - were assessed using five-point Likert scales. Demographic and dialysis-related information included age, sex, dialysis modality, dialysis vintage, CV disease history, daily pill burden and omega-3 (n-3) use. The complete questionnaire is provided in Appendix S1 (English version) and Appendix S2 (German version).

Data Collection and Processing

Survey data were collected using paper-based questionnaires (German version) distributed at nine dialysis centers, which were located across multiple Austrian federal states and included three university hospitals, four regional public hospitals, and two peripheral dialysis units. Participating centers were recruited on a voluntary basis through the Hemodialysis Working Group of the ÖGN, aiming to include a range of care settings across several Austrian federal states. Centers were not randomly sampled and represent a subset of all Austrian dialysis facilities. The survey was conducted anonymously, and no identifying information was collected at any stage of data collection or analysis. Completed questionnaires were entered into an electronic data file using Microsoft Excel Version 16.106 (Microsoft Corp., Redmond, WA, USA). Data were checked for completeness and internal consistency prior to analysis. Only questionnaires containing a valid response to the primary item assessing willingness to take fish oil supplementation under full reimbursement were analyzed.

Statistical Analyses

Continuous variables are reported as median [quartile 1; quartile 3] and categorical variables are reported as counts and percentages. Responses to the willingness item were summarized descriptively for each cost scenario.

For the primary outcome (willingness to take 4 g fish oil daily if recommended by the treating nephrologist and fully reimbursed), responses were dichotomized as “willing” (summarizing “Yes, definitely”, “Probably yes”) and “unwilling” (summarizing “Probably not”, “No, definitely not”), and the proportion of “willing” was reported with 95% confidence intervals (CI). This dichotomized outcome was chosen as the primary endpoint to improve clinical interpretability of effect estimates and to align with common practice in acceptability research.

To examine within-participant changes in willingness across different cost scenarios (full reimbursement, partial self-payment, full self-payment), binary mixed-effects logistic regression modelled willingness (dichotomized) as the dependent variable, and cost scenario as a fixed effect. Random intercepts were added for participants to account for repeated responses provided by the same participant across the three cost scenarios. In sensitivity analyses, the original four-category willingness outcome (ordered from “No, definitely not” to “Yes, definitely”) was analyzed using an ordinal mixed-effects logistic regression model. Results are presented as odds ratios (OR) with 95% CI.

Associations between willingness under full reimbursement and attitudinal factors were examined using logistic regression models. Attitudinal factors (measured on 5-point Likert scales) were entered as numeric predictors, assuming a linear effect across response categories, to allow estimation of a single, easily interpretable OR per 1-point increase on the attitudinal scale. In sensitivity analyses, the original four-category willingness outcome (ordered from “No, definitely not” to “Yes, definitely”) was analyzed using ordinal logistic regression models. Results are presented as ORs with 95% CI.

No formal correction for multiple testing was applied, as analyses were considered exploratory and hypothesis-generating. Missing data were handled using a complete-case approach for each analysis. Statistical analyses were performed using R Version 4.5.2 (R Foundation for Statistical Computing, Vienna, Austria).

Table S1: Patient characteristics, stratified by willingness to take fish oil supplementation under full reimbursement.

| **Variable** | **Overall** | **Willing** | **Unwilling** |
| --- | --- | --- | --- |
| n | n=525 | n=417 | n=108 |
| Age, years¹⁵ | 69.0 [58.0;78.0] | 68.0 [56.0;77.0] | 70.0 [63.0;80.0] |
| Sex⁸ |  |  |  |
| female | 196 (37.9%) | 156 (38.0%) | 40 (37.7%) |
| male | 320 (61.9%) | 255 (62.0%) | 65 (61.3%) |
| diverse | 1 (0.2%) | 0 (0.0%) | 1 (0.9%) |
| Dialysis vintage¹⁰ |  |  |  |
| < 1 year | 85 (16.5%) | 76 (18.6%) | 9 (8.5%) |
| 1-3 years | 180 (35.0%) | 144 (35.2%) | 36 (34.0%) |
| 3-5 years | 104 (20.2%) | 76 (18.6%) | 28 (26.4%) |
| > 5 years | 146 (28.3%) | 113 (27.6%) | 33 (31.1%) |
| History of cardiovascular disease (self-reported)¹⁵ |  |  |  |
| yes | 225 (44.1%) | 186 (46.0%) | 39 (36.8%) |
| no | 248 (48.6%) | 190 (47.0%) | 58 (54.7%) |
| I don’t know | 37 (7.3%) | 28 (6.9%) | 9 (8.5%) |
| Daily pill count¹³ |  |  |  |
| 0-5 | 100 (19.5%) | 77 (18.9%) | 23 (21.9%) |
| 6-10 | 201 (39.3%) | 154 (37.8%) | 47 (44.8%) |
| 11-15 | 142 (27.7%) | 121 (29.7%) | 21 (20.0%) |
| more than 15 | 69 (13.5%) | 55 (13.5%) | 14 (13.3%) |
| Current omega-3 use¹¹ |  |  |  |
| Yes | 47 (9.1%) | 43 (10.5%) | 4 (3.8%) |
| No | 458 (89.1%) | 360 (87.8%) | 98 (94.2%) |
| I don’t know | 9 (1.8%) | 7 (1.7%) | 2 (1.9%) |
| Past omega-3 experience¹⁶ |  |  |  |
| Yes, and I tolerated them well | 105 (20.6%) | 102 (25.2%) | 3 (2.9%) |
| Yes, but I did not tolerate them well | 14 (2.8%) | 6 (1.5%) | 8 (7.7%) |
| No | 363 (71.3%) | 279 (68.9%) | 84 (80.8%) |
| I don’t know / don’t remember | 27 (5.3%) | 18 (4.4%) | 9 (8.7%) |

Legend to Table S1: Continuous variables are presented as median [quartile 1; quartile 3]; categorical variables as n (%). Willingness was defined as responding "Yes, definitely" or "Probably yes" to the primary willingness item under full reimbursement. Superscripts indicate the number of participants with missing values for the respective variable.

Table S2: Distribution of willingness to take fish oil supplementation across three cost scenarios.

| **Variable** | **Full reimbursement n (%)** | **95% CI** | **Partial self-payment n (%)** | **95% CI** | **Full self-payment n (%)** | **95% CI** |
| --- | --- | --- | --- | --- | --- | --- |
| Willingness (binary) | n=525 |  | n=524 |  | n=522 |  |
| Willing | 417 (79.4%) | 75.8–82.7% | 295 (56.3%) | 52.0–60.5% | 199 (38.1%) | 34.1–42.4% |
| Unwilling | 108 (20.6%) | 17.3–24.2% | 229 (43.7%) | 39.5–48.0% | 323 (61.9%) | 57.6–65.9% |
| Willingness (categorical) | n=525 |  | n=524 |  | n=522 |  |
| Yes, definitely | 233 (44.4%) | 40.2–48.7% | 135 (25.8%) | 22.2–29.7% | 74 (14.2%) | 11.4–17.4% |
| Probably yes | 184 (35.0%) | 31.1–39.2% | 160 (30.5%) | 26.7–34.6% | 125 (23.9%) | 20.5–27.8% |
| Probably not | 70 (13.3%) | 10.7–16.5% | 146 (27.9%) | 24.2–31.9% | 171 (32.8%) | 28.9–36.9% |
| No, definitely not | 38 (7.2%) | 5.3–9.8% | 83 (15.8%) | 13.0–19.2% | 152 (29.1%) | 25.4–33.2% |

Legend to Table S2: Willingness is presented both as a binary outcome (willing: "Yes, definitely" or "Probably yes"; unwilling: "Probably not" or "No, definitely not") and as the original four-category response distribution. Values are presented as n (%) with 95% confidence intervals (CI).

Table S3: Association between cost scenario and willingness to take fish oil supplementation: results from an ordinal mixed-effects logistic regression model (sensitivity analysis).

| **Variable** | **OR** | **95% CI** | **p value** |
| --- | --- | --- | --- |
| Partial self-payment vs full reimbursement | 0.15 | 0.11 – 0.20 | <0.001 |
| Full self-payment vs full reimbursement | 0.03 | 0.02 – 0.04 | <0.001 |

Legend to Table S3: Results are presented as odds ratios (OR) with 95% confidence intervals (CI). The model included cost scenario as a fixed effect (reference: full reimbursement) and a random intercept for participant to account for repeated measures. The four-category willingness response (ordered from "No, definitely not" to "Yes, definitely") served as the outcome.

Table S4: Associations between patient characteristics, attitudinal factors, and willingness to take fish oil supplementation under full reimbursement: results from univariable logistic regression models.

| **Variable (n=525)** | **Scale** | **OR** | **95% CI** | **p value** |
| --- | --- | --- | --- | --- |
| Expected effectiveness of fish oil⁸ | Numerical (Likert scale) | 2.97 | 2.35–3.74 | <0.001 |
| Trust in treating nephrologist¹⁰ | Numerical (Likert scale) | 2.11 | 1.78–2.51 | <0.001 |
| Perceived cardiovascular risk²¹ | Numerical (Likert scale) | 1.56 | 1.31–1.86 | <0.001 |
| Perceived pill burden¹² | Numerical (Likert scale) | 0.72 | 0.62–0.84 | <0.001 |
| Concerns about side effects¹⁰ | Numerical (Likert scale) | 0.68 | 0.58–0.80 | <0.001 |
| Understood information on fish oil¹⁶ | Numerical (Likert scale) | 1.35 | 1.16–1.58 | <0.001 |
| Understood study purpose²² | Numerical (Likert scale) | 1.46 | 1.24–1.71 | <0.001 |
| Age, years¹⁵ | Numerical | 0.98 | 0.96–1.00 | 0.015 |
| Sex⁸: male | Categorical (reference: female) | 1.01 | 0.65–1.56 | 0.979 |
| Sex⁸: diverse | Categorical (reference: female) | 0.00 | 0.00–Inf | 0.978 |
| Dialysis vintage (per category increase)¹⁰ | Ordinal (per category increase) | 0.80 | 0.65–0.98 | 0.033 |
| History of cardiovascular disease (self-reported)¹⁵: no | Categorical (reference: yes) | 0.69 | 0.44–1.08 | 0.104 |
| History of cardiovascular disease (self-reported)¹⁵: I don’t know | Categorical (reference: yes) | 0.65 | 0.29–1.49 | 0.311 |
| Daily pill count (per category increase)¹³ | Ordinal (per category increase) | 1.16 | 0.92–1.46 | 0.206 |
| Current omega-3 use¹¹: No | Categorical (reference: Yes) | 0.34 | 0.12–0.97 | 0.045 |
| Current omega-3 use¹¹: I don’t know | Categorical (reference: Yes) | 0.33 | 0.05–2.12 | 0.241 |
| Past omega-3 experience¹⁶: Yes, but I did not tolerate them well | Categorical (reference: Yes, and I tolerated them well) | 0.02 | 0.00–0.11 | <0.001 |
| Past omega-3 experience¹⁶: No | Categorical (reference: Yes, and I tolerated them well) | 0.10 | 0.03–0.32 | <0.001 |
| Past omega-3 experience¹⁶: I don’t know / don’t remember | Categorical (reference: Yes, and I tolerated them well) | 0.06 | 0.01–0.24 | <0.001 |

Legend to Table S4: Results are presented as odds ratios (OR) with 95% confidence intervals (CI) and p-values derived from univariable binary logistic regression models. Attitudinal factors were modeled as numeric predictors per 1-point increase on a 5-point Likert scale. Dialysis vintage and daily pill count were modeled as ordinal predictors per category increase. Categorical variables are presented relative to a reference category, which is indicated in parentheses alongside each variable. Superscripts indicate the number of participants with missing values for the respective variable.

Table S5: Distribution of attitudinal factor responses stratified by willingness to take fish oil supplementation under full reimbursement.

| **Variable** | **Overall** | **95% CI)** | **Willing** | **95% CI)** | **Unwilling** | **95% CI** |
| --- | --- | --- | --- | --- | --- | --- |
| n | n=525 |  | n=417 |  | n=108 |  |
| Expected effectiveness of fish oil⁸ |  |  |  |  |  |  |
| 1 | 44 (8.5%) | 6.4–11.2% | 14 (3.4%) | 2.0–5.6% | 30 (28.6%) | 20.8–37.8% |
| 2 | 52 (10.1%) | 7.8–13.0% | 27 (6.6%) | 4.5–9.4% | 25 (23.8%) | 16.7–32.8% |
| 3 | 168 (32.5%) | 28.6–36.6% | 133 (32.3%) | 27.9–36.9% | 35 (33.3%) | 25.0–42.8% |
| 4 | 131 (25.3%) | 21.8–29.3% | 119 (28.9%) | 24.7–33.4% | 12 (11.4%) | 6.7–18.9% |
| 5 | 122 (23.6%) | 20.1–27.4% | 119 (28.9%) | 24.7–33.4% | 3 (2.9%) | 1.0–8.1% |
| Trust in treating nephrologist¹⁰ |  |  |  |  |  |  |
| 1 | 45 (8.7%) | 6.6–11.5% | 22 (5.4%) | 3.6–8.0% | 23 (22.1%) | 15.2–31.0% |
| 2 | 32 (6.2%) | 4.4–8.6% | 15 (3.6%) | 2.2–5.9% | 17 (16.3%) | 10.5–24.6% |
| 3 | 82 (15.9%) | 13.0–19.3% | 50 (12.2%) | 9.4–15.7% | 32 (30.8%) | 22.7–40.2% |
| 4 | 119 (23.1%) | 19.7–26.9% | 103 (25.1%) | 21.1–29.5% | 16 (15.4%) | 9.7–23.5% |
| 5 | 237 (46.0%) | 41.8–50.3% | 221 (53.8%) | 48.9–58.5% | 16 (15.4%) | 9.7–23.5% |
| Perceived cardiovascular risk²¹ |  |  |  |  |  |  |
| 1 | 59 (11.7%) | 9.2–14.8% | 32 (8.0%) | 5.7–11.1% | 27 (26.0%) | 18.5–35.1% |
| 2 | 93 (18.5%) | 15.3–22.1% | 68 (17.0%) | 13.6–21.0% | 25 (24.0%) | 16.8–33.1% |
| 3 | 143 (28.4%) | 24.6–32.5% | 118 (29.5%) | 25.2–34.1% | 25 (24.0%) | 16.8–33.1% |
| 4 | 86 (17.1%) | 14.0–20.6% | 74 (18.5%) | 15.0–22.6% | 12 (11.5%) | 6.7–19.1% |
| 5 | 123 (24.4%) | 20.9–28.3% | 108 (27.0%) | 22.9–31.6% | 15 (14.4%) | 8.9–22.4% |
| Perceived pill burden¹² |  |  |  |  |  |  |
| 1 | 125 (24.4%) | 20.9–28.3% | 105 (25.7%) | 21.7–30.1% | 20 (19.2%) | 12.8–27.8% |
| 2 | 66 (12.9%) | 10.2–16.0% | 60 (14.7%) | 11.6–18.4% | 6 (5.8%) | 2.7–12.0% |
| 3 | 105 (20.5%) | 17.2–24.2% | 91 (22.2%) | 18.5–26.5% | 14 (13.5%) | 8.2–21.3% |
| 4 | 80 (15.6%) | 12.7–19.0% | 65 (15.9%) | 12.7–19.8% | 15 (14.4%) | 8.9–22.4% |
| 5 | 137 (26.7%) | 23.1–30.7% | 88 (21.5%) | 17.8–25.8% | 49 (47.1%) | 37.8–56.6% |
| Concerns about side effects¹⁰ |  |  |  |  |  |  |
| 1 | 150 (29.1%) | 25.4–33.2% | 128 (31.1%) | 26.9–35.8% | 22 (21.2%) | 14.4–30.0% |
| 2 | 81 (15.7%) | 12.8–19.1% | 73 (17.8%) | 14.4–21.7% | 8 (7.7%) | 3.9–14.4% |
| 3 | 125 (24.3%) | 20.8–28.2% | 103 (25.1%) | 21.1–29.5% | 22 (21.2%) | 14.4–30.0% |
| 4 | 78 (15.1%) | 12.3–18.5% | 61 (14.8%) | 11.7–18.6% | 17 (16.3%) | 10.5–24.6% |
| 5 | 81 (15.7%) | 12.8–19.1% | 46 (11.2%) | 8.5–14.6% | 35 (33.7%) | 25.3–43.2% |
| Understood information on fish oil¹⁶ |  |  |  |  |  |  |
| 1 | 60 (11.8%) | 9.3–14.9% | 43 (10.5%) | 7.9–13.9% | 17 (17.0%) | 10.9–25.5% |
| 2 | 55 (10.8%) | 8.4–13.8% | 41 (10.0%) | 7.5–13.3% | 14 (14.0%) | 8.5–22.1% |
| 3 | 111 (21.8%) | 18.4–25.6% | 79 (19.3%) | 15.8–23.4% | 32 (32.0%) | 23.7–41.7% |
| 4 | 107 (21.0%) | 17.7–24.8% | 90 (22.0%) | 18.3–26.3% | 17 (17.0%) | 10.9–25.5% |
| 5 | 176 (34.6%) | 30.6–38.8% | 156 (38.1%) | 33.6–42.9% | 20 (20.0%) | 13.3–28.9% |
| Understood study purpose²² |  |  |  |  |  |  |
| 1 | 40 (8.0%) | 5.9–10.6% | 29 (7.2%) | 5.1–10.1% | 11 (11.0%) | 6.3–18.6% |
| 2 | 47 (9.3%) | 7.1–12.2% | 32 (7.9%) | 5.7–11.0% | 15 (15.0%) | 9.3–23.3% |
| 3 | 93 (18.5%) | 15.3–22.1% | 62 (15.4%) | 12.2–19.2% | 31 (31.0%) | 22.8–40.6% |
| 4 | 99 (19.7%) | 16.4–23.4% | 79 (19.6%) | 16.0–23.8% | 20 (20.0%) | 13.3–28.9% |
| 5 | 224 (44.5%) | 40.2–48.9% | 201 (49.9%) | 45.0–54.7% | 23 (23.0%) | 15.8–32.2% |

Legend to Table S5: Values are presented as n (%) with 95% confidence intervals (CI). Attitudinal factors were measured on 5-point Likert scales (1–5). Willingness was defined as responding "Yes, definitely" or "Probably yes" to the primary willingness item under full reimbursement. Superscripts indicate the number of participants with missing values for the respective variable.

Table S6: Associations between attitudinal factors and willingness to take fish oil supplementation under full reimbursement: results from univariable ordinal logistic regression models (sensitivity analysis).

| **Variable (n=525)** | **Scale** | **OR** | **95% CI** | **p value** |
| --- | --- | --- | --- | --- |
| Expected effectiveness⁸ (n=517) | Numerical (Likert scale) | 3.19 | 2.67–3.81 | <0.001 |
| Trust in nephrologist¹⁰ (n=515) | Numerical (Likert scale) | 2.09 | 1.81–2.42 | <0.001 |
| Perceived CV risk²¹ (n=504) | Numerical (Likert scale) | 1.55 | 1.35–1.77 | <0.001 |
| Pill burden¹² (n=513) | Numerical (Likert scale) | 0.72 | 0.64–0.81 | <0.001 |
| Side-effect concerns¹⁰ (n=515) | Numerical (Likert scale) | 0.59 | 0.52–0.67 | <0.001 |
| Understood fish oil info¹⁶ (n=509) | Numerical (Likert scale) | 1.39 | 1.23–1.58 | <0.001 |
| Understood study purpose²² (n=503) | Numerical (Likert scale) | 1.48 | 1.29–1.68 | <0.001 |

Legend to Table S6: Results are presented as odds ratios (OR) with 95% confidence intervals (CI) and p-values. Attitudinal factors were modeled as numeric predictors per 1-point increase on a 5-point Likert scale. The four-category willingness response (ordered from "No, definitely not" to "Yes, definitely") served as the outcome. The OR represents the odds of being in a higher willingness category per 1-point increase in each predictor.

Figure S1: Willingness to take fish oil supplementation across different Likert scale values of attitudinal factors.

Legend to Figure S1: Stacked bar charts showing the proportion of participants willing (black) or unwilling (grey) to take fish oil supplementation (4 g daily) under full reimbursement, stratified by response level (1–5) for each of seven Likert scale items. Response levels 1 to 5 reflect a 5-point Likert scale. Sample sizes per response level (n) are displayed above each bar.

Figure S2: Associations between attitudinal factors and willingness to take fish oil supplementation under full reimbursement.

Legend to Figure S2: Forest plot showing odds ratios (OR) and 95% confidence intervals derived from univariable logistic regression models assessing the association between attitudinal factors (modeled per 1-point increase on a 5-point scale) and willingness to take fish oil supplementation (4 g daily) if fully reimbursed. ORs are displayed on a logarithmic scale to ensure symmetric visual representation of effects in both directions and to prevent distortion arising from the multiplicative nature of the OR scale. The dashed vertical line indicates an OR of 1 (no association). OR greater than 1 indicate higher odds of willingness, whereas OR below 1 indicate lower odds of willingness.

Figure S3: Associations between patient characteristics and willingness to take fish oil supplementation under full reimbursement.

Legend to Figure S3: Forest plot showing odds ratios (OR) and 95% confidence intervals derived from univariable logistic regression models assessing the association between demographic and clinical characteristics and willingness to take 4 g fish oil daily if fully reimbursed. Continuous predictors (age) were modeled per 10-year increase; ordinal predictors (dialysis vintage, daily pill count) were modeled per category increase; categorical predictors are shown relative to their reference category. OR are displayed on a logarithmic scale to ensure symmetric visual representation of effects in both directions and to prevent distortion arising from the multiplicative nature of the OR scale. The dashed vertical line indicates an OR of 1 (no association). OR greater than 1 indicate higher odds of willingness, whereas OR below 1 indicate lower odds of willingness

Figure S4: Associations between attitudinal factors and willingness to take fish oil supplementation under full reimbursement: ordinal logistic regression (sensitivity analysis).

Legend to Figure S4: Forest plot showing odds ratios (OR) and 95% confidence intervals derived from univariable ordinal logistic regression models assessing the association between attitudinal constructs (modeled per 1-point increase on a 5-point Likert scale) and the four-category willingness outcome (ordered from "No, definitely not" to "Yes, definitely"). OR represent the odds of being in a higher willingness category per 1-point increase in each predictor. OR are displayed on a logarithmic scale to ensure symmetric visual representation of effects in both directions and to prevent distortion arising from the multiplicative nature of the OR scale. The dashed vertical line indicates an OR of 1 (no association). OR greater than 1 indicate higher odds of willingness, whereas OR below 1 indicate lower odds of willingness.

## Appendix S1: Full Questionnaire (English Version)

**Survey on the Intake of Fish Oil Capsules**

for Dialysis Patients

Questionnaire Version 1.2 – 12^th^ Dec 2025

Thank you for taking the time! Participation is **voluntary** and **anonymous** and takes about **7-10 minutes**. Please turn the page – the information and questionnaire begin on the next page.

Section 1: Information and Consent

You are invited to take part in an **anonymous** survey. This survey explores how dialysis patients feel about a potential intake of fish-oil capsules.

**Why are we conducting this survey?**

A recent major study in dialysis patients (“PISCES study”) has caused **considerable excitement** in the dialysis world. For the first time, daily **fish-oil capsules** were shown to markedly **reduce the risk of serious cardiovascular events** (such as heart attacks or strokes). These findings are important, as cardiovascular disease remains the leading cause of death in dialysis patients.

Fish-oil capsules are not currently part of standard therapy, but the positive results have sparked exciting discussions about whether fish-oil capsules could become a new efficient therapy in the future.

Before any decisions can be made, it is essential to understand how patients themselves view this option. Your opinion is therefore central to shaping future care.

If you wish to participate, please check the box below:

☐ **I confirm that I have read the information and voluntarily participate in the anonymous survey.**

Section 2: Brief Information on Fish Oil

Please read the following information:

This survey concerns fish-oil capsules that would be taken as four capsules per day. This is a hypothetical scenario – you would not actually take the capsules.

Fish oil is a dietary supplement and not a conventional medication. Fish oil contains omega-3 fatty acids, which are also found in fish such as salmon or mackerel. In a large study with dialysis patients (“PISCES study”), people who took 4 g of fish-oil capsules daily had a significantly lower risk of serious cardiovascular events – about 40% lower than those who did not receive fish oil.

Possible side effects of fish oil capsules include mild gastrointestinal discomfort, belching, or a “fishy” taste. In some studies in people without kidney disease, an increased risk of atrial fibrillation and bleeding has been reported; however, in the recent PISCES study (including only dialysis patients), the risk of bleeding and atrial fibrillation was not increased.

You will now be asked several questions. Please check the option that applies.

**Again:** this is only a hypothetical scenario. You will not actually take fish-oil capsules.

1. How well informed do you feel about the purpose of this survey?

1 = not informed at all, 5 = very well informed

1 ☐ 2 ☐ 3 ☐ 4 ☐ 5 ☐

2. How well informed do you feel about the effects and side effects of fish-oil capsules?

1 = not informed at all, 5 = very well informed

1 ☐ 2 ☐ 3 ☐ 4 ☐ 5 ☐

Section 3: Key Questions on Willingness to Take Fish Oil

3. If your doctor recommended taking fish-oil capsules and the costs were fully covered by health insurance, would you take them?

☐ Yes, definitely

☐ Probably yes

☐ Probably not

☐ No, definitely not

4. What if you had to pay a small amount yourself (e.g., €20 per month)? Would you then take the fish-oil capsules?

☐ Yes, definitely

☐ Probably yes

☐ Probably not

☐ No, definitely not

5. What if you had to pay the full cost yourself (approx. €40 per month)? Would you take the fish-oil capsules?

☐ Yes, definitely

☐ Probably yes

☐ Probably not

☐ No, definitely not

Section 4: Questions on Your Perception and Views

Please indicate how much you **agree** with the following statements:

6. I consider my personal risk for cardiovascular disease (e.g., heart attack, stroke) to be high.

(1 = strongly disagree, 5 = strongly agree)

1 ☐ 2 ☐ 3 ☐ 4 ☐ 5 ☐

7. I think that fish oil capsules could reduce my cardiovascular risk.

(1 = strongly disagree, 5 = strongly agree)

1 ☐ 2 ☐ 3 ☐ 4 ☐ 5 ☐

8. I trust the recommendations of my doctor.

(1 = strongly disagree, 5 = strongly agree)

1 ☐ 2 ☐ 3 ☐ 4 ☐ 5 ☐

9. Four additional capsules per day would be a burden for me.

(1 = strongly disagree, 5 = strongly agree)

1 ☐ 2 ☐ 3 ☐ 4 ☐ 5 ☐

10. I am concerned about possible side effects of fish oil capsules.

(1 = strongly disagree, 5 = strongly agree)

1 ☐ 2 ☐ 3 ☐ 4 ☐ 5 ☐

Section 5: Information About You

This information helps us to better understand the results. The survey remains **anonymous**!

11. Age: _______
12. Gender:	☐ female	☐ male	☐ diverse
13. Dialysis modality:	☐ Hemodialysis	☐ Peritoneal dialysis
14. How long have you been on dialysis?

☐ < 1 year

☐ 1–3 years

☐ 3–5 years

☐ > 5 years

15. Have you ever been diagnosed with a cardiovascular disease (e.g., heart attack, stroke, heart failure)?

☐ yes	☐ no	☐ I don’t know

16. How many tablets or capsules do you usually take per day?

☐ 0–5	☐ 6–10	☐ 11-15	☐ more than 15

17. Are you currently taking fish oil or omega-3 capsules?

☐ Yes ☐ No ☐ I don’t know

18. Have you taken fish oil or omega-3 capsules in the past?

☐ No

☐ Yes, and I tolerated them well

☐ Yes, but I did not tolerate them well

☐ I don’t know / don’t remember

**The survey ends here – Thank you for your participation!**

## Appendix S2: Full Questionnaire (German Version)

**Umfrage zur Einnahme von Fischöl-Kapseln**

für Dialysepatient:innen

Fragebogen Version 1.2 – 12.12.2025

Vielen Dank, dass Sie sich Zeit nehmen! Die Teilnahme ist **freiwillig** und **anonym** und dauert etwa **7-10 Minuten.** Bitte umblättern – die Informationen und der Fragebogen beginnen auf der Rückseite.

Themenblock 1: Information und Einwilligung

Sie werden eingeladen, an einer **anonymen** Umfrage teilzunehmen. In dieser Befragung geht es darum, wie Patient:innen zu einer möglichen Einnahme von Fischöl stehen.

**Wieso machen wir diese Umfrage?**

Eine **große neue Studie** mit Dialysepatient:innen („PISCES-Studie“) hat für viel Aufmerksamkeit gesorgt. Zum ersten Mal konnte gezeigt werden, dass die tägliche Einnahme von **Fischölkapseln** das **Risiko für schwere Herz-Kreislauf-Ereignisse** (wie Herzinfarkt oder Schlaganfall) **deutlich senken** kann. Diese Ergebnisse sind besonders wichtig, da Herz-Kreislauf-Erkrankungen nach wie vor die häufigste Todesursache bei Dialysepatient:innen sind.

Fischölkapseln gehören derzeit nicht zur Standardtherapie. Diese neuen Ergebnisse haben aber dazu geführt, dass viele Ärzt:innen darüber nachdenken, ob Fischölkapseln in Zukunft eine **zusätzliche Schutzmaßnahme** sein könnten.

Damit gute Entscheidungen für die Zukunft getroffen werden können, möchten wir wissen, wie Patient:innen über diese Möglichkeit denken. Ihre Meinung ist daher sehr wichtig.

Wenn Sie teilnehmen möchten, kreuzen Sie bitte das folgende Kästchen an:

☐ **Ich bestätige, dass ich die Informationen gelesen habe und freiwillig an der anonymen Umfrage teilnehme.**

Themenblock 2: Kurzinformation zum Fischöl

Bitte lesen Sie die folgenden Informationen:

Es geht in dieser Umfrage um Fischöl-Kapseln, die man täglich (4 Stück) schlucken würde. Es handelt sich dabei um eine hypothetische Frage – Sie würden die Kapseln nicht wirklich einnehmen.

Fischöl ist ein Nahrungsergänzungsmittel und kein Medikament im herkömmlichen Sinne. Fischöl enthält sogenannte Omega-3-Fettsäuren. Diese kommen auch in Fisch vor, zum Beispiel in Lachs oder Makrele. In einer großen Studie mit Dialysepatient:innen („PISCES-Studie“) hatten Menschen, die jeden Tag 4g Fischöl-Kapseln nahmen, ein deutlich geringeres Risiko für schwere Herz-Kreislauf-Probleme. Das Risiko war etwa 40 % niedriger als bei Menschen, die keine Fischöl-Kapseln bekamen.

Mögliche Nebenwirkungen von Fischöl-Kapseln können sein: leichte Magen-Darm-Beschwerden; Aufstoßen oder „fischiger“ Geschmack. In einigen Studien bei nierengesunden Menschen wurde ein erhöhtes Risiko für Vorhofflimmern und Blutungen beschrieben - in der neuen „PISCES-Studie“ (nur Dialysepatient:innen) war das Risiko für Blutungen und Vorhofflimmern aber normal.

Es erwarten Sie nun mehrere Fragen. Bitte kreuzen Sie jeweils das Zutreffende an.

**Nochmals zur Erinnerung:** Es handelt sich nur um eine hypothetische Einnahme von Fischöl-Kapseln, Sie werden diese nicht wirklich einnehmen!

1. Wie gut fühlen Sie sich über den Zweck dieser Umfrage informiert?

(1 = gar nicht informiert, 5 = sehr gut informiert)

1 ☐ 2 ☐ 3 ☐ 4 ☐ 5 ☐

2. Wie gut fühlen Sie sich über Wirkungen und Nebenwirkungen von Fischöl-Kapseln informiert?

(1 = gar nicht informiert, 5 = sehr gut informiert)

1 ☐ 2 ☐ 3 ☐ 4 ☐ 5 ☐

Themenblock 3: Kernfragen zur Einnahmebereitschaft

3. Wenn Ihre Ärztin / Ihr Arzt Ihnen die Einnahme von Fischöl-Kapseln empfehlen würde und die Kosten vollständig von der Krankenkasse übernommen würden, würden Sie es einnehmen?

☐ Ja, auf jeden Fall

☐ Eher ja

☐ Eher nein

☐ Nein, auf keinen Fall

4. Wie wäre es, wenn Sie für die Fischöl-Kapseln einen kleinen Teil (z. B. 20 € pro Monat) selbst bezahlen müssten? Würden Sie die Fischöl-Kapseln dann einnehmen?

☐ Ja, auf jeden Fall

☐ Eher ja

☐ Eher nein

☐ Nein, auf keinen Fall

5. Wie wäre es, wenn Sie die Kosten für die Fischöl-Kapseln vollständig selbst bezahlen müssten (z. B. ca. 40€ pro Monat)? Würden Sie die Fischöl-Kapseln dann einnehmen?

☐ Ja, auf jeden Fall

☐ Eher ja

☐ Eher nein

☐ Nein, auf keinen Fall

Themenblock 4: Fragen zu Ihrer Einschätzung bzw. Sichtweise

Bitte kreuzen Sie an, wie sehr Sie den folgenden Aussagen **zustimmen**:

6. Ich schätze mein persönliches Risiko für Herz-Kreislauf-Erkrankungen (z. B. Herzinfarkt, Schlaganfall) als hoch ein.

(1 = stimme überhaupt nicht zu, 5 = stimme voll zu)

1 ☐ 2 ☐ 3 ☐ 4 ☐ 5 ☐

7. Ich denke, dass Fischöl-Kapseln mein Herz-Kreislauf-Risiko senken könnten.

(1 = stimme überhaupt nicht zu, 5 = stimme voll zu)

1 ☐ 2 ☐ 3 ☐ 4 ☐ 5 ☐

8. Ich vertraue den Empfehlungen meiner Ärztin / meines Arztes.

(1 = stimme überhaupt nicht zu, 5 = stimme voll zu)

1 ☐ 2 ☐ 3 ☐ 4 ☐ 5 ☐

9. Vier zusätzliche Kapseln pro Tag wären für mich belastend.

(1 = stimme überhaupt nicht zu, 5 = stimme voll zu)

1 ☐ 2 ☐ 3 ☐ 4 ☐ 5 ☐
10. Ich mache mir Sorgen wegen möglicher Nebenwirkungen von Fischöl-Kapseln.

(1 = stimme überhaupt nicht zu, 5 = stimme voll zu)

1 ☐ 2 ☐ 3 ☐ 4 ☐ 5 ☐

Themenblock 5: Angaben zu Ihrer Person

Diese Angaben helfen uns, die Ergebnisse besser einzuordnen. Die Umfrage bleibt **anonym**!

11. Alter: ________
12. Geschlecht:	☐ weiblich ☐ männlich ☐ divers
13. Dialysemodalität:	☐ Hämodialyse	☐ Peritonealdialyse
14. Wie lange sind Sie ungefähr dialysepflichtig?

☐ weniger als 1 Jahr

☐ 1-3 Jahre

☐ 3-5 Jahre

☐ mehr als 5 Jahre

15. Wurde bei Ihnen jemals eine Herz-Kreislauf-Erkrankung festgestellt (z. B. Herzinfarkt, Schlaganfall, Herzschwäche)?

☐ ja ☐ nein ☐ weiß ich nicht

16. Wie viele Tabletten oder Kapseln nehmen Sie üblicherweise pro Tag ein?

☐ 0–5 ☐ 6–10 ☐ 11-15	☐ mehr als 15

17. Nehmen Sie derzeit Fischöl- oder Omega-3-Kapseln ein?

☐ ja ☐ nein ☐ weiß ich nicht

18. Haben Sie Fischöl- oder Omega-3-Kapseln früher schon einmal genommen?

☐ Nein

☐ Ja, und ich habe sie gut vertragen

☐ Ja, aber ich habe sie nicht gut vertragen

☐ Weiß ich nicht / erinnere mich nicht

**Die Umfrage ist an dieser Stelle abgeschlossen – nochmals vielen Dank für Ihre Teilnahme!**
