# Supplementary material for: Willingness to take fish-oil supplementation among hemodialysis patients: a multicenter survey following the PISCES trial
Source: Clin Kidney J. 2026 Aug 12;19(9):sfag269. doi: 10.1093/ckj/sfag269 (PMC13542717; doi:10.1093/ckj/sfag269)
Supplement: Supplementary file 1 [file sfag269_supplemental_files.zip › FigureS1.pdf]

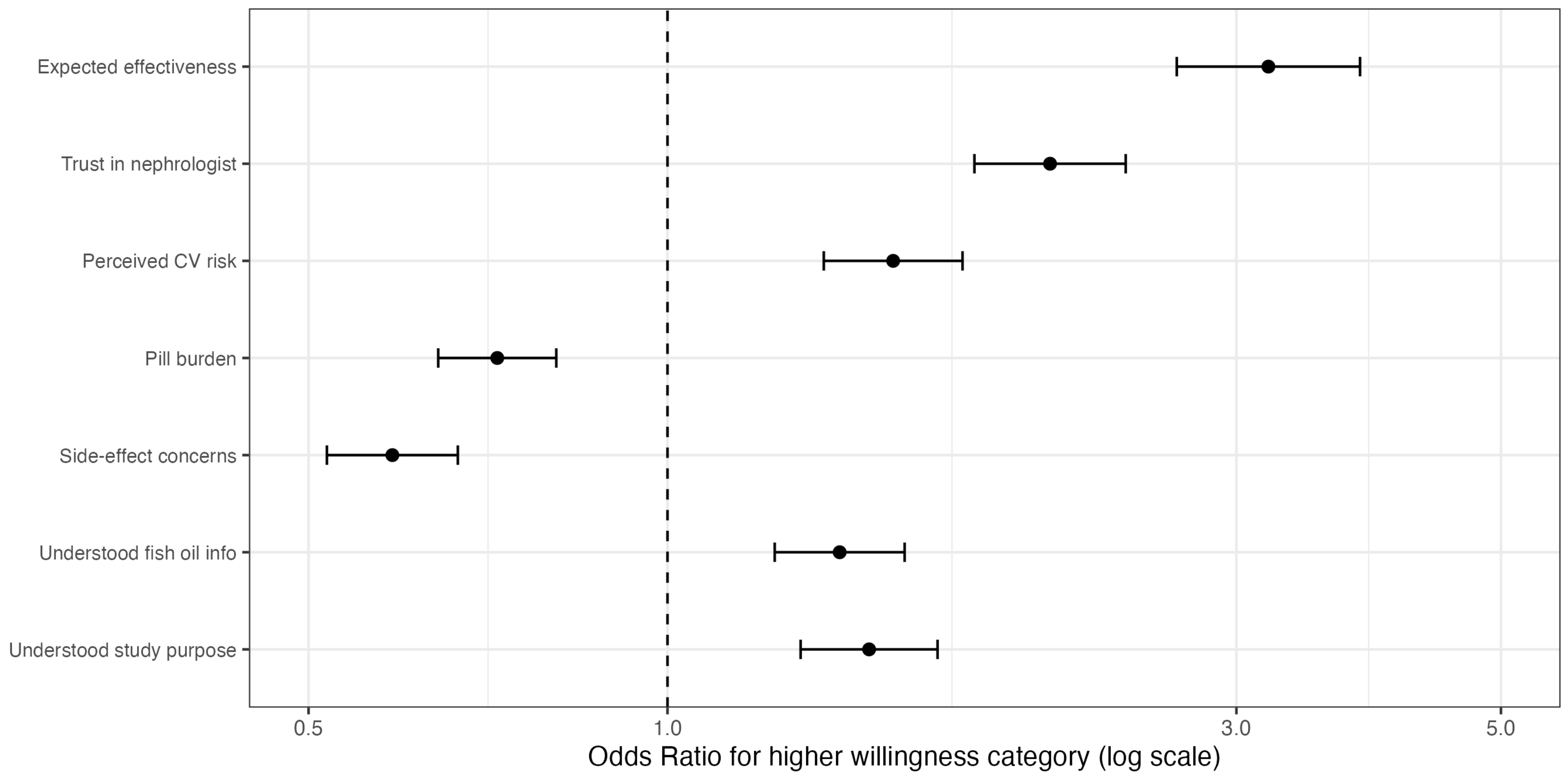
Expected effectiveness
Trust in nephrologist
Perceived CV risk
Pill burden
Side-effect concerns
Understood fish oil info
Understood study purpose
0.5
1.0
3.0
5.0
Odds Ratio for higher willingness category (log scale)
